# BacAv, a new free online platform for clinical back-averaging

**DOI:** 10.1016/j.cnp.2019.12.001

**Published:** 2020-01-25

**Authors:** Felipe Vial, Sanaz Attaripour, Patrick McGurrin, Mark Hallett

**Affiliations:** a Human Motor Control Section, National Institute of Neurological Disorders and Stroke, National Institutes of Health, Bethesda, MD, USA; b Facultad de Medicina Clínica Alemana Universidad del Desarrollo, Santiago, Chile

**Keywords:** Back-average, Bereitschaftspotential, Myoclonus

## Abstract

**Objective:**

The back-average technique is very useful to study the relation between the activity in the cortex and the muscles. It has two main clinical applications, Bereitschaftspotential (BP) recording and myoclonus studies. The BP is a slow wave negativity originating in the supplementary motor cortex and premotor cortex that precedes voluntary movements. This wave also precedes involuntary movements in functional movement disorders (FMD), and it can be used as a helpful diagnostic tool. For the myoclonus studies, the back-average technique is very important to help localizing the source of the myoclonus. The hardware needed to do BP or myoclonus studies is standard and available in any electrophysiology lab, but there are not many software solutions to do the analysis. In this article together with describing the methodology that we use for recording clinical BPs and myoclonus, we present BacAv, an online free application that we developed for the purpose of doing back-average analysis.

**Methods:**

BacAv was developed in “R” language using Rstudio, a free integrated development environment. The recommended parameters for the data acquisition for BP recording and myoclonus studies are given in this section.

**Results:**

The platform was successfully developed, is able to read txt files, look for muscle bursts, segment the data, and plot the average. The parameters of the algorithm that look for the muscle bursts can be adapted according to the characteristics of the dataset.

**Conclusion:**

We have developed software for clinicians who do not have sophisticated equipment to do back-averaging.

**Significance:**

This tool will make this useful analysis method more available in a clinical environment.

## Highlights

• Back-averaging is a useful technique to correlate the activity of the motor cortex with a muscle jerk.
• Back-averaging is a tool for the diagnosis of conditions such as functional movement disorders and cortical myoclonus.
• BacAv, a new online platform, can be used for back-average analysis.

## 1 Introduction

Muscle contraction depends on motor neuron activation, and motor neurons depend on spinal, brainstem and cortical inputs. For the diagnosis of certain medical conditions, it is helpful to know the characteristics of the relationship between cortical activity and muscle activity. Clinical neurophysiological techniques can simultaneously record muscle and cortical activity and then their relationship can be evaluated.

With surface electromyography (EMG) even without further data processing, it is easy to differentiate if a muscle is resting or active. If it is active, characteristics such as burst duration and amplitude can be measured. Brain activity, on the other hand, is much more complicated to characterize. When recording electroencephalography (EEG), different neuron populations are firing at all times producing mixtures of local field potentials creating a constant background activity (Purdon et al., 2015). The muscle-related activity that is being looked for may be hidden in this background.

The idea of the back-average technique is to help find this hidden information in the EEG. The way it is done is by segmenting the simultaneous EEG-EMG recording in equally sized windows in relation to the event of interest in the EMG including the time before the beginning of the EMG burst as well as after it. The segments are then averaged. All the EEG information that is time-locked to the event of interest in the EMG, will survive the average, the rest will be canceled out (Tassinari et al., 1998).

One clinical application of the back-average is to look for Bereitschaftspotential (BP) in functional (psychogenic) movement disorders (FMD). The BP is a movement-related cortical potential generally used for detecting the participation of the ‘voluntary motor system’ in the generation of movements. In patients with FMD, the pathways normally used to produce voluntary movements are also used in the generation of involuntary abnormal movements. Therefore, a BP is usually identified prior to the abnormal involuntary movements of FMD (Czarnecki and Hallett, 2012). Separation of functional movement disorders and organic movement disorders merely based on clinical assessment can be challenging and it is not uncommon that making a diagnosis of FMD in a patient becomes a matter of controversy among clinicians. Therefore, in an attempt to improve the diagnostic certainty, Gupta and Lang (2009) proposed adding “laboratory-supported definite” as a new category of clinical diagnostic classification to the diagnostic criteria of functional movement disorders (Gupta and Lang, 2009). In the context of abrupt or jerky abnormal movements, the laboratory support can be provided by capturing BP via back-averaging of the EEG preceding the movements. In this way, it is possible to distinguish functional and organic myoclonus (Terada et al., 1995). Back-averaging can also be applied in distinguishing functional and organic tics as preceding the organic tics either there is no BP or only a small BP (Karp et al., 1996, Vial et al., 2019).

In the case of myoclonus, the back-average technique can help to demonstrate the presence of an EEG potential preceding the myoclonus, giving evidence of a cortical origin. The correct anatomic localization of the myoclonic activity is helpful not only for a more accurate diagnosis, but it can also have therapeutic implications.

Despite how useful this technique can be, it is only available in a few places and is mostly used for research purposes. The hardware needed to record the data is standard and available in any clinical electrophysiology lab, but the software needed to do the back-average is not widely available. That and maybe also the fact that manually analyzing the data is time-consuming and laborious are the reasons that this method is underutilized.

Our purpose was to develop a free online platform that would serve as a tool for clinicians to do the back-average analysis. In this article we are also going to describe our method in the recording techniques for BP, myoclonic studies, and also the analysis as can be done in this new platform.

## 2 Methods

### 2.1 Online platform development:

This platform was coded in “R” language using Rstudio, a free and open-source integrated development environment (IDE) for R. It was developed as an analysis tool that will read txt. files. The idea was that clinicians will be able to record with the hardware and software available in their institution and then transform the data to txt. file to be analyzed in this platform.

### 2.2 Clinical recording of BP in FMD patients

#### 2.2.1 Parameters for the recording

##### 2.2.1.1 EMG

The EMG recording is done with surface electrodes. The selection of muscles being recorded for both functional movement and myoclonus studies is critical for good analysis. In case of doubt, it is always a good practice to record several muscles in the area involved in the movement and their neighboring muscles. Before placing the electrodes, the skin area has to be prepared with an abrasive substance in order to reduce impedances ideally below 10 Ω (Hermens et al., 2000). This is important to reduce movement artifacts. The EMG frequency content is mainly between 10 Hz and 250 Hz; therefore, a sampling rate of at least 1 kHz should be used. Movement artifact is generally below 20 Hz. A filtering window between 20 and 300 Hz will capture most of the EMG signal avoiding the low-frequency movement artifacts (Nilsson et al., 1993).

##### 2.2.1.2 EEG

The BP is a very broad potential widely distributed over the scalp regardless of the site of movement (Shibasaki and Hallett, 2006), therefore only one EEG electrode on the contralateral sensory-motor region (C3-C4) or at the vertex in case of axial or lower extremities’ movements may be enough to record it (although we usually record from both sides). In regard to the reference, we use the contralateral mastoid.

The filtering parameters are critical. Standard EEG recording is done with a high pass of 1 Hz and a low pass of 70 Hz (Sinha et al., 2016). The BP is a very slow wave that can start up to 2 s before the movement onset (Shibasaki and Hallett, 2006) which will give a frequency of 0.25 Hz so it would be filtered if these standard parameters are used. In our practice we have good results using a high pass of 0.01 Hz and a low pass of 50 Hz.

#### 2.2.2 Behavioral recording

Two sets of recording have to be done. First, spontaneous involuntary movements have to be recorded. In general, we try to record at least 40–50 involuntary movements (but sometimes it is difficult to record this many movements, especially when the frequency of the movements is very low). The rationale behind that number is that about 20 trials are usually enough to see a BP. By recording twice that many, it will be possible to split the trials and average separately. The persistence of BP in the split data confirms the result.

Then, voluntary movements should be recorded. This is useful as a positive control of the quality of the technique. For the voluntary movement recording, a simple movement in a region not involved in the involuntary movement may be recorded such as an abduction of the opponens pollicis brevis. Another approach is to ask the subject to mimic the involuntary movements. This second approach has the advantage of studying the same muscle group involved in the involuntary movement, but it has the complication that during the recording there might be voluntary and involuntary movements, so these movements would need to be separated (the subject can be asked to let the technician know immediately if an involuntary movement happened during the voluntary mimicked movements, so that movement can be marked and excluded from the mimicked movement analysis). In either case, the subject has to be instructed to “decide” to do movements at any time during the recording. The only restriction is that the movement has to be done with a frequency slower than every 5 s in order to have a good BP.

### 2.3 Clinical recording of patients with myoclonus

#### 2.3.1 Parameters for the recording

##### 2.3.1.1 EMG

The EMG parameters (filters and sampling rate) are the same as described for the BP technique. The selection of muscles depends on the phenomenology, but in general, for distal myoclonus is helpful to record pairs of agonist–antagonist muscles (because the co-contraction of the agonist–antagonist pair is a sign that the burst is centrally driven). When the myoclonus is more axial, is important to record muscles representing different myotomes and cranial nerves if there are involved for better source localization.

##### 2.3.1.2 EEG

In this case, the standard EEG filter parameters (1–70 Hz) can be used, unless there is clinical suspicion of FMD in which case is better to use the parameters described in the BP section. In our lab, we prefer to always record with the described “BP parameters” and then if it is necessary, narrow the parameters in the post-processing because this cannot be done in the opposite direction.

In regard to the electrodes, is important to record the contralateral motor cortex (C3, C4) but also go more frontal (F3, F4) as the myoclonic depolarization is sometimes better seen in that region (Wilkins et al., 1984).

#### 2.3.2 Behavioral recording

The idea is to record the maximum number of myoclonic bursts, so the recording conditions depend on how the myoclonus is evoked. In our lab, we always record the subject when the muscles involved are at rest and then during posture and different actions, again depending on how the myoclonus is evoked.

A summary of the recording conditions can be found in Table 1.

**Table 1:** Summary of the recommended recording parameters for BP and myoclonus.

	Bereitschaftspotential	Myoclonus
EMG filters	10–250 Hz	10–250 Hz
EEG filters	0.01–50 Hz	1–70 Hz
Sampling rate	At least 1000 Hz	At least 1000 Hz
Behavioral recording	Record involuntary and voluntary movements.	Record involuntary movements during rest, posture and action depending on how the myoclonus is evoked.

## 3 Results

**Fig. 1:**
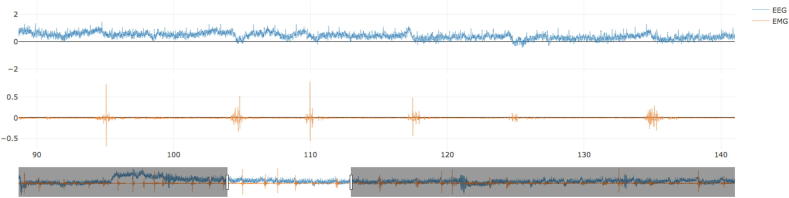
First step, load the data. Once the data are loaded both channels (EEG and EMG) will be displayed and can be inspected.

The online platform, named BacAv, was successfully developed and it can be found in https://electrophysiology.shinyapps.io/BacAv/. In order to use BacAV, the data have to be transformed to txt. files in such a way that each channel is a column separated by space (an example of a dataset is given as Supplementary Material (Supplement 1)). There should be no headers in the txt. file columns. Once the data is in that format, it can be loaded and visualized. The columns corresponding to the EMG and EEG channel to be used (the 3rd and 4th column, respectively, in the Supplementary example), together with the sampling rate (1000 Hz in the Supplementary example) at which the data were acquired has to be specified (Fig. 1).

Once the data is visualized, it is possible to move to the “Time Domain” window. By clicking on “RUN” button, the EMG data will be analyzed by an algorithm that will look for muscle bursts and put markers. To do this, the data are rectified, and the amplitudes are re-scaled from 0 to 1 in order to increase the algorithm sensibility for finding muscle bursts.

**Fig. 2:**
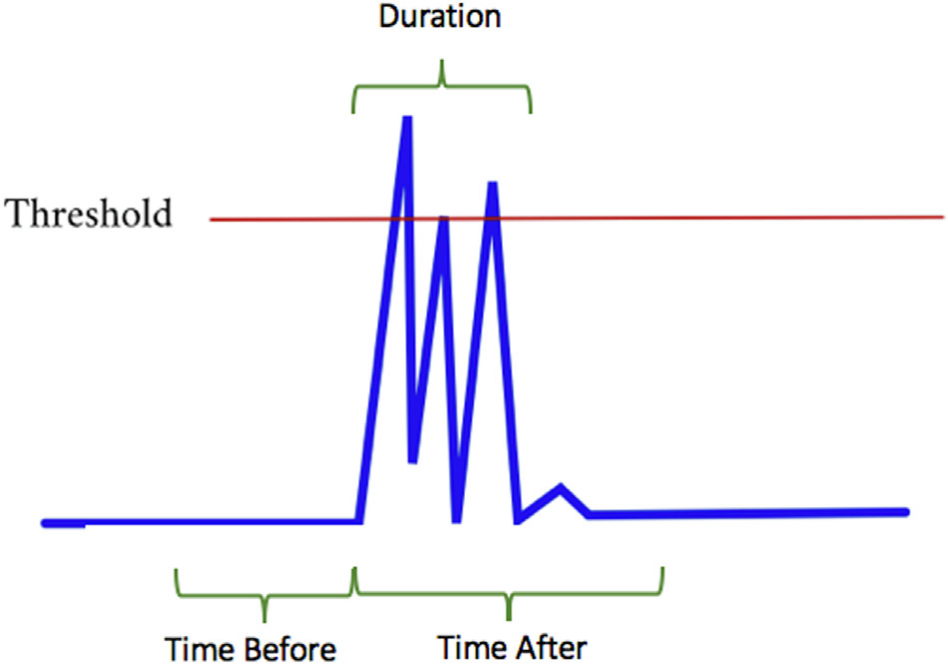
Second step, adjust parameters. The figure shows a representation of the parameters that have to be adjusted in order to let the algorithm look for the muscle bursts.

The following are the adjustable parameters that the algorithm will use (Fig. 2):

- Threshold: Value between 0 and 1 over what the muscle activity has to be in order to be considered a candidate burst.
- Time before: Window of time (in seconds) before a muscle activity exceeding the threshold.
- Time after: Window of time (in seconds) after a muscle activity exceeding the threshold.
- Amplitude before: The mean amplitude in the “Time before” window has to be lower than the value specified in this parameter.
- Amplitude after: The mean amplitude in the “Time after” window has to be higher than the value specified in this parameter.
- Burst duration: Window of time (in seconds) after which no new muscle burst can be marked.

**Fig. 3:**
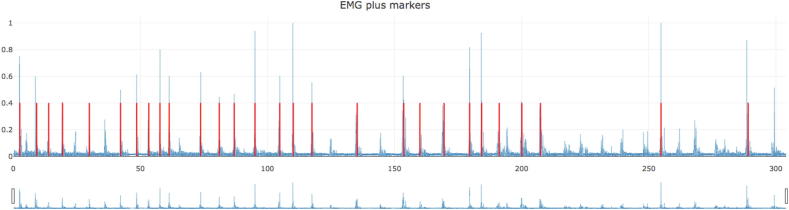
Third step, visualize markers. After the “RUN” button is press, the algorithm will place markers in the muscle bursts. If the result is not good, the parameters can be adjusted, and the algorithm run again.

In the upper right corner, the number of markers would be indicated. The parameters can be changed, and the algorithm can be run as many times as needed in order to get a representative number of bursts. The EMG plot can be zoomed in with the mouse and there is a scrolling bar below the plot (Fig. 3).

There are two other parameters in this window that will be used for the segmentation:

- Window: Window in seconds indicating the length of the segments.
- Onset: This will set the position of the time-point “0″, the onset of the muscle burst.

**Fig. 4:**
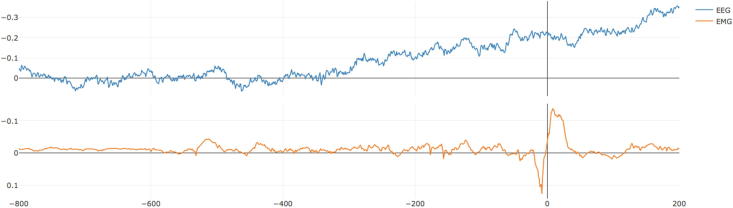
Fourth step, visualize average. In this step, the data will be segmented according to the markers placed in the previous step and then averaged. The segment used for baseline correction can be adjusted. In this example is possible to observe a nice Bereitschaftspotential preceding the muscle burst.

Once the markers are placed, the next step is to move to the “Average” window. In this window, there is one parameter to set, the length in seconds starting from the beginning of the segments that will be used for baseline correction. After hitting the “RUN” button, an average of the EEG and EMG in relation to the previously obtained markers will be displayed (Fig. 4).

**Fig. 5:**
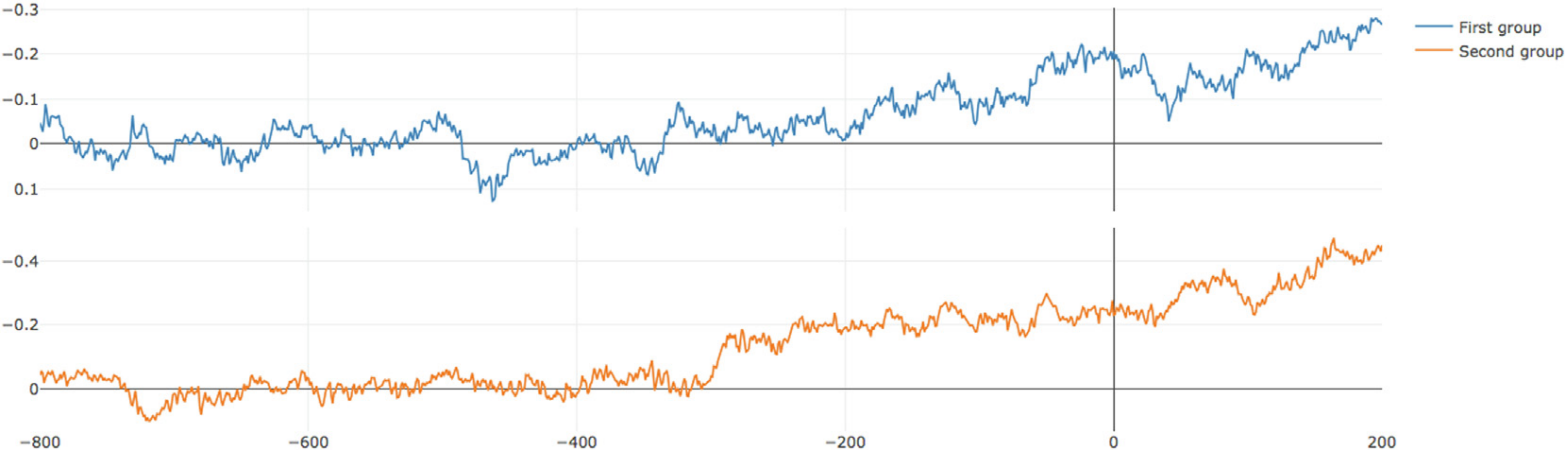
Fifth step, reorder and split. In this window the segments will be randomly divided into two groups, and the average of the two groups will be displayed. This can be run many times in order to look for consistency.

The next step is the “Reorder and split” window. In this window, segments will be randomly re-ordered and split into two groups and the EEG average of the two groups will be plotted. This can be run several times and the idea is to look for consistency in the shape of the wave (Fig. 5).

## 4 Discussion

BacAv is a free online platform that will allow users to do back-averaging. It can take files in a txt. format, look for markers in the EMG, and then average. Furthermore, it is possible to split the data and reorder it, to look for consistency of the observed wave.

**Fig. 6:**
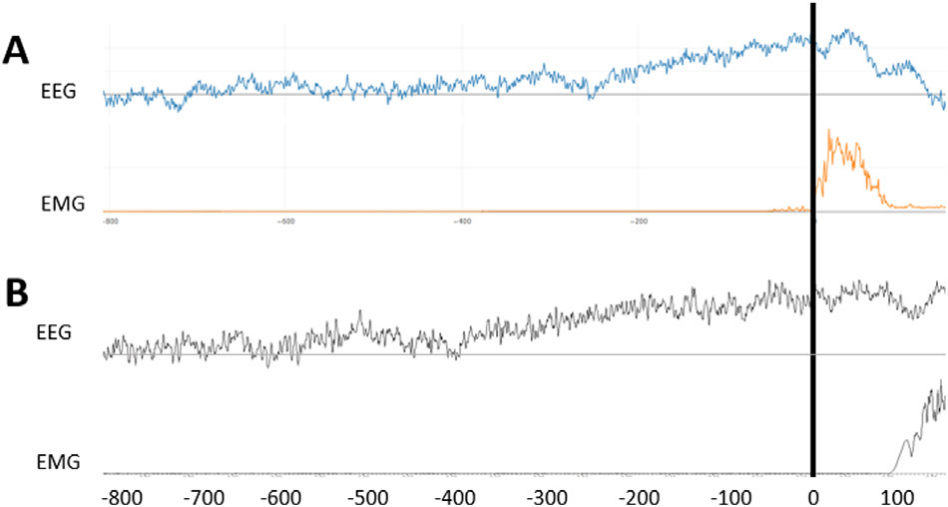
Comparison between BacAv and Spike software. Both traces correspond to the same EEG-EMG recording with voluntary movements. A: Analysis done with BacAv, B: Analysis done with Spike.

The alternative method that we use in the lab for this type of study is to analyze the data with Spike (Cambridge Electronic Design) software. This software allows to place manually the markers at the beginning of the bursts. This may be more precise but it takes a lot of time and applying the correct parameters we have found that the results are comparable (Fig. 6).

The recording of both BP and myoclonic potentials requires paying careful attention to the filter parameters. It is also very important to do a good selection of the involved muscles and also to record both voluntary and involuntary movements.

In this article, we described the technique for BP and myoclonic study recordings, as well as releasing a new platform for back-averaging that we hope will be useful for clinicians.

## Conflict of Interest Statement

Dr. Felipe Vial received salary support through a research project funded by Cala Health Inc. (875 Mahler Rd #168, Burlingame, CA 94010).

Dr. Hallett may accrue revenue on US Patent #6,780,413 B2 (Issued: August 24, 2004): Immunotoxin (MAB-Ricin) for the treatment of focal movement disorders, and US Patent #7,407,478 (Issued: August 5, 2008): Coil for Magnetic Stimulation and methods for using the same (H-coil); in relation to the latter, he has received license fee payments from the NIH (from Brainsway) for licensing of this patent. He is on the Medical Advisory Boards of CALA Health, Brainsway, and Cadent. He is on the Editorial Board of approximately 15 journals and receives royalties and/or honoraria from publishing from Cambridge University Press, Oxford University Press, Springer, and Elsevier. Dr. Hallett's research at the NIH is largely supported by the NIH Intramural Program. Supplemental research funds have been granted by Allergan for studies of methods to inject botulinum toxins, Medtronic, Inc. for a study of DBS for dystonia, and CALA Health for studies of a device to suppress tremor.
